# SRF617 Is a Potent Inhibitor of CD39 with Immunomodulatory and Antitumor Properties

**DOI:** 10.4049/immunohorizons.2200089

**Published:** 2023-05-23

**Authors:** Michael C. Warren, Stephan Matissek, Matthew Rausch, Marisella Panduro, Richard J. Hall, Austin Dulak, David Brennan, Sonia Das Yekkirala, Secil Koseoglu, Ricard Masia, Yu Yang, Navamallika Reddy, Robert Prenovitz, Jamie Strand, Tauqueer Zaidi, Erik Devereaux, Célia Jacoberger Foissac, John Stagg, Benjamin H. Lee, Pamela Holland, Vito J. Palombella, Andrew C. Lake

**Affiliations:** * Surface Oncology, Inc., Cambridge, MA; † Université de Montréal, Centre de Recherche du Centre hospitalier de l’Université de Montréal, Montreal, Quebec, Canada

## Abstract

CD39 (ENTPD1) is a key enzyme responsible for degradation of extracellular ATP and is upregulated in the tumor microenvironment (TME). Extracellular ATP accumulates in the TME from tissue damage and immunogenic cell death, potentially initiating proinflammatory responses that are reduced by the enzymatic activity of CD39. Degradation of ATP by CD39 and other ectonucleotidases (e.g., CD73) results in extracellular adenosine accumulation, constituting an important mechanism for tumor immune escape, angiogenesis induction, and metastasis. Thus, inhibiting CD39 enzymatic activity can inhibit tumor growth by converting a suppressive TME to a proinflammatory environment. SRF617 is an investigational, anti-CD39, fully human IgG4 Ab that binds to human CD39 with nanomolar affinity and potently inhibits its ATPase activity. In vitro functional assays using primary human immune cells demonstrate that inhibiting CD39 enhances T-cell proliferation, dendritic cell maturation/activation, and release of IL-1β and IL-18 from macrophages. In vivo, SRF617 has significant single-agent antitumor activity in human cell line–derived xenograft models that express CD39. Pharmacodynamic studies demonstrate that target engagement of CD39 by SRF617 in the TME inhibits ATPase activity, inducing proinflammatory mechanistic changes in tumor-infiltrating leukocytes. Syngeneic tumor studies using human CD39 knock-in mice show that SRF617 can modulate CD39 levels on immune cells in vivo and can penetrate the TME of an orthotopic tumor, leading to increased CD8^+^ T-cell infiltration. Targeting CD39 is an attractive approach for treating cancer, and, as such, the properties of SRF617 make it an excellent drug development candidate.

## Introduction

The purine nucleoside adenosine plays a critical role in dampening innate and adaptive immune responses under inflammatory conditions (1). In contrast, high levels of extracellular ATP resulting from tissue damage or immunogenic cell death can initiate proinflammatory responses (2). Extracellular adenosine accumulates in cancerous tissues through ATP degradation and constitutes an important mechanism for tumor immune escape, angiogenesis induction, and metastasis (3). Early studies demonstrated that adenosine could inhibit CD3^+^ T cell and NK cell function and that endogenous adenosine levels in tumors would be sufficient to mediate immunosuppression (4). Other studies have shown that high levels of ATP can act as a potent chemoattractant for innate immune cells and mediate robust inflammation, leading to adaptive immune response activation (2). Thus, both extracellular adenosine and ATP play important roles in cancer maintenance and progression. Inhibition of extracellular adenosine accumulation while maintaining high levels of ATP in the tumor microenvironment (TME) may therefore have anticancer therapeutic effects.

Reduction of ATP and subsequent increase of extracellular adenosine production in the TME depend on the concerted enzymatic activity of two ectonucleotidases: CD39 (ENTPD1) and CD73 (NT5E). CD39 catalyzes the hydrolysis of ATP to AMP, and CD73 hydrolyzes AMP into adenosine. Inhibition or loss of CD39 has been shown to have antitumor effects in multiple preclinical studies. Notably, growth of MC38 colon cancer cells and B16 melanoma hepatic metastatic tumors was significantly inhibited in CD39 knockout mice compared with wild-type mice (5). Interestingly, hepatic metastatic growth was reduced in chimeric mice reconstituted with CD39^−/−^ bone marrow–derived cells, suggesting that CD39 on immune cells alone contributes to tumor growth. A complementary study reported CD39 transgenic mice overexpressing CD39 to have larger, more rapidly growing metastatic tumors after injection with CT26 colorectal cells (6). Emerging clinical study data demonstrate the importance of extracellular adenosine generation and signaling in human cancer progression and that targeting this pathway can benefit cancer treatment. Prolonged disease stabilization in humans has been observed in clinical studies of adenosine receptor inhibitors and CD73 inhibitors (targets downstream from CD39 in the adenosine pathway) as monotherapies and in combination with other therapies, including chemotherapy regimens and immune checkpoint inhibition (7–12). Taken together, these observations demonstrate the potential for targeting CD39 in cancer, further validating the importance of the extracellular adenosinergic pathway in promoting tumor growth.

SRF617 is a fully human anti-CD39 Ab that prevents CD39-mediated conversion of ATP to AMP and phosphate. We demonstrate that SRF617 is a potent inhibitor of CD39 enzymatic activity and can affect immune cell function in vitro and in vivo. We also demonstrate through mouse models that CD39 inhibition in vivo has antitumor effects while lowering adenosine levels and increasing inflammation in the TME. SRF617 is currently being evaluated in clinical studies across multiple tumor types as a monotherapy and in combinations (NCT04336098 and NCT05177770).

## Materials and Methods

### Generation of anti-human CD39 Ab

The CD39 binding portions of SRF617 were initially discovered using a selection campaign in yeast starting with de novo synthetic human Ab diversity (for review of diversity design and library assembly, see Xu et al. [13]). A library totaling 10^10^ in size was initiated to discover Abs with CD39 binding specificity (Adimab). Using a biotinylated form of recombinant CD39 together with MACS on the first two rounds followed by FACS, successive rounds of enrichment narrowed the Ab library diversity to those with the desired CD39 specificity (for a review of techniques, see Chao et al. [14]). This selection was followed by subsequent rounds of affinity maturation of selected Abs with anti-CD39 enzymatic properties, leading to the discovery of SRF617.

### Cell lines and tissues

The MOLP-8 cell line was obtained from Deutsche Sammlung von Mikroorganismen und Zellkulturen and maintained in 10% FBS in RPMI plus 1% penicillin/streptomycin. Human buffy coat and whole blood were obtained from healthy donors at Research Blood Components, LLC. Human B cells were isolated from buffy coat using RosetteSep Human B Cell Enrichment Cocktail (STEMCELL Technologies) followed by Ficoll-Paque (GE Healthcare) density gradient purification centrifugation. Monocytes were isolated from healthy human donor blood using RosetteSep Human Monocyte Enrichment Cocktail (STEMCELL Technologies). HEK293, CT26, and H520 cell lines were purchased from American Type Culture Collection and maintained in 10% FBS in RPMI plus 1% penicillin/streptomycin.

### Abs and tool reagents

SRF617 Ab was generated by ProBioGen. PE-anti-human CD39 Ab A1 clone (PE-CD39.A1) positive control, anti-huIgG4 isotype control Ab, PE-huIgG Fc secondary Ab, rat anti-murine CD39 (Duha59), and purified anti-human CD16, CD32, and CD64 Abs were purchased from BioLegend. DNP IgG4 single mutant isotype control Ab was generated at WuXi Biologics.

### Ab binding to recombinant human, cynomolgus monkey, and mouse CD39

#### Human and cynomolgus monkey CD39 binding to SRF617

All kinetic analyses on the ForteBio Octet QK^e^ were performed using 1× kinetics buffer (10× kinetics buffer [ForteBio] diluted in PBS, pH 7.4) as the diluent for the SRF617 ligand and the CD39 analytes. Kinetic analysis by biolayer interferometry (BLI) for recombinant human and cynomolgus monkey CD39 was performed by capturing SRF617 ligand at 1 µg/ml for 5 min using anti-human Fc Octet biosensors followed by a single baseline step of 60 s in 1× kinetics buffer. SRF617-captured biosensors were dipped in wells containing 0 to 1.25 µg/ml of either recombinant human or cynomolgus monkey CD39 analytes for 10 min of association followed by 15 min of dissociation in 1× kinetics buffer. A no-analyte SRF617-captured reference biosensor was included to compensate for the natural dissociation of the captured SRF617 during the analysis. Sample plates were on an orbital shaker (1000 rpm) at 25°C during the analysis.

#### Mouse CD39 binding to SRF617 and Duha59 (positive control Ab)

Kinetic analysis by BLI for recombinant mouse CD39 was performed by covalently capturing 20 µg/ml of either SRF617 or Duha59 ligand in 10 mM acetate, pH 6.0, using the amine-reactive second-generation kit and AR2G biosensors (ForteBio). Following a 120-s baseline step, a 10-min association of the SRF617- or Duha59-captured biosensors was done by dipping into wells containing 0 to 1.25 µg/ml recombinant mouse CD39 followed by a 20-min dissociation in 1× kinetics buffer. A no-analyte SRF617 or Duha59-captured reference biosensor was included to compensate for any dissociation of captured Abs during analysis. Sample plates were on an orbital shaker (1000 rpm) at 25°C during analysis.

#### Data analysis

All data from 0 to 1.25 µg/ml analyte concentrations were subtracted from the no-analyte (no recombinant CD39) reference biosensor and smoothed, aligned, and fitted using Data Analysis software version 10.0.3.7 (ForteBio) using a 1:1 global binding model.

### Determination of Ab binding to cell surface CD39

Cells were plated at 2 × 10^5^ cells/well in a 96-well plate (Thermo Fisher). MOLP-8 cells were treated with a Human TruStain FcX block (BioLegend). B cells were treated with an Fc block mixture containing anti-human CD16, CD32, and CD64 Abs. Cells were incubated with 1.5 ng/ml to 30 μg/ml of either SRF617 or huIgG4 isotype control Ab. PE-huIgG4 Fc secondary Ab was used for detection. Cells were analyzed by flow cytometry, and geometric mean fluorescence intensity was calculated using FlowJo software.

### Cellular enzymatic inhibition

MOLP-8 (5000 cells/well) or RBC-lysed whole blood was added to a 96-well plate (Corning) and allowed to settle for 30 min, after which 0.5 ng/ml to 30 μg/ml of either SRF617 or isotype control Ab was incubated with the cells in duplicate for 60 min, followed by 25 μM ATP (Thermo Fisher) for an additional 60 min. During the incubation, cellular CD39 catalyzed the hydrolysis of ATP to AMP, thereby releasing free phosphate. Malachite green dye (Enzo Life Sciences) was added to supernatants to react with free phosphate, resulting in increased absorbance at 620 nm. Control wells did not contain any Ab. One set of control wells contained cells and ATP added at the beginning of the enzymatic reaction (high absorbance, 0% inhibition); the other set contained cells and ATP added at the end of the reaction (low absorbance, 100% inhibition). Absorbance values were used to calculate the percentage inhibition of ATP hydrolysis and the IC_50_. Assays were performed in phosphate-free buffer.

### CD4^+^ T-cell proliferation

Cryopreserved CD4^+^ T cells isolated from PBMCs of three individual donors using a CD4^+^ isolation kit (STEMCELL Technologies) were pooled and prestained with proliferation detection dye using a CellTrace Violet Cell Proliferation Kit (Thermo Fisher). Prestained cells were added to a 96-well plate (Costar) and incubated with 10 μg/ml of SRF617 or isotype control Ab, 250 μM ATP (Thermo Fisher), and anti-CD3/anti-CD28 activating Dynabeads (Thermo Fisher) for 3 d. Human TruStain FcX was used to block unwanted Fc receptor staining, and anti-CD4 allophycocyanin conjugated (BD Pharma) and anti-CD39 PE conjugated (BD Pharma) were used for gating cells during flow cytometry. Gating cells for analysis showed that 14.6% of CD4^+^ cells were CD39^+^. Cells were analyzed for proliferation. For calculation of the proliferation index, each discrete generation of proliferation received a weighted factor; increased proliferation resulted in a larger factor. The percentage of CD4^+^ T cells in each discrete generational gate was multiplied by this weighted factor. The formula for the weighted factor was 2 ⁁ (Generation # − 1). Thus, for Generation 1, 2 ⁁ (1, 1) = 1, and for Generation 4, 2 ⁁ (4, 1) = 8. Proliferation index values were plotted in GraphPad Prism software against each Ab calculation to create a concentration (*x*-value) versus proliferation index (*y*-value) plot. A four-parameter nonlinear fit was achieved using GraphPad Prism software.

### Dendritic cell maturation

Monocyte-derived dendritic cells (DCs) were generated by differentiating monocytes from unpurified buffy coats of three individual human donors for 6 d with 40 ng/ml IL-4 (R&D Systems) and 200 ng/ml granulocyte M-CSF (R&D Systems). In the CD86 and HLA-DR isotype expression experiment, cells from all three donors were treated with 10 μg/ml SRF617 or isotype control Ab and incubated in the presence or absence of 300 μM ATP (Thermo Fisher) for 24 h in 96-well plates (Corning). Upon completion of the experiment, cells were incubated with Human TruStain FcX (BioLegend) to prevent nonspecific Ab binding and with LIVE/DEAD Fixable Near-Infrared Dead Cell Stain (Thermo Fisher) to exclude dead cells from downstream FACS analysis. To measure CD86 expression, cells were stained with a PE-conjugated CD86 Ab (BioLegend) and an allophycocyanin-conjugated HLA-DR Ab; the readout was the geometric mean of CD86 and HLA-DR. Cells were analyzed on a BD LSRFortessa X-20. Mean ± SD values for CD86 and HLA-DR geometric mean were calculated. Raw data values were exported from FlowJo. Data were plotted, and an unpaired *t* test was performed between isotype control Ab and SRF617 treatment for each donor and ATP condition using GraphPad Prism software.

In the IL-16 and IL-12/IL-23p40 secretion experiment, monocyte-derived DCs from donors were treated with 10 μg/ml SRF617 or isotype control Ab and incubated in the presence or absence of 300 μM ATP for 48 h in 96-well plates. Cell supernatants were collected and transferred to a Meso Scale Discovery (MSD) plate, and the MSD protocol was followed to measure IL-16 and IL-12/IL-23p40 secretion. Plates were read on a Meso Quick Plex SQ120.

### IL-1β and IL-18 release from macrophages

Monocytes were plated in a 96-well flat-bottomed plate and incubated in RPMI medium containing 10% FBS, 1% penicillin/streptomycin, and 400 ng/ml granulocyte M-CSF (R&D Systems) for 6 d in a 37°C incubator. On day 6, 0.1 × 10^6^ cells/well were replated in a 96-well flat-bottomed plate and allowed to attach overnight in a 37°C incubator. Macrophages were then treated with 10 µg/ml isotype control Ab or SRF617 for 1 h, followed by 10 ng/ml LPS (Sigma-Aldrich) (*Escherichia coli* serotype 0111:B4) for 3 h and 0, 500, or 1000 µM ATP (Thermo Fisher) for 2 h at 37°C. Supernatant was removed and analyzed for IL-1β and IL-18 by MSD multiplex assay.

### CD39 expression on isolated human B cells after SRF617 treatment

Cryopreserved human PBMCs were thawed in RPMI plus 10% FBS, counted, and plated in a 96-well round-bottomed plate at 200,000 cells per well. Cells were placed in a 37°C incubator and rested overnight. Cells were treated with SRF617 at a range of concentrations, returned to the incubator, and rested for 2 d. An additional set of cells was treated just prior to analysis to serve as a T = 0 control to rule out the possibility of Ab cross-blocking. Cells were washed once with flow buffer (PBS plus 2% FBS); 100 μl of FcX blocking solution was added to all wells; and 100 μl of flow staining mixture containing LIVE/DEAD Fixable Near-IR (780) (Thermo Fisher), anti-CD19, anti-CD39, and anti-CD45 was added to all wells. After incubating at 4°C for 30 min, 100 μl of flow buffer was added as a quench. Cells were washed twice with flow buffer, fixed with 1% paraformaldehyde, and analyzed on a BD LSRFortessa X-20.

### In vivo studies

All animal studies were carried out in strict accordance with the recommendations in the Guide for the Care and Use of Laboratory Animals of the National Institutes of Health under protocols approved by the Charles River Accelerator and Development Lab (protocol number CR-008) or Mispro Biotech Services (protocol number 2020-SUR-2) institutional animal care and use committees, and all efforts were made to minimize suffering. Orthotopic pancreatic cancer experiments with human CD39 knock-in (hCD39 KI) mice were performed in accordance with the Canadian laws for animal protection and were approved by the Institutional Animal Protection Committee (Comité Institutionnel de Protection des Animaux, Centre hospitalier de l’Université de Montréal, Montreal, QC, Canada).

All animals completed the study as planned, and behavior and health observations were normal. No animals or data points were excluded from the calculations. Data were analyzed using GraphPad Prism software (version 6.07). Mean ± SEM values were calculated, and treated groups were compared with the isotype control Ab group using two-way ANOVA with Dunnett’s multiple comparison test (MOLP-8 tumor growth inhibition) or two-tailed unpaired *t* test (H520, hCD39 KI, MOLP-8 immunohistochemistry [IHC], and KPC). A *p* value <0.05 was considered statistically significant.

### MOLP-8 xenograft model

#### Tumor growth inhibition

Forty-three 5- to 7-wk-old female SCID mice were injected s.c. with 1 × 10^7^ MOLP-8 cells and randomized via matched distribution method using Study Director software into four unblinded groups when tumors reached mean volumes of 100 mm^3^. Groups in individual cages were treated in the same order i.p. twice per week with either 30 (*n* = 11), 10 (*n* = 10), or 3 (*n* = 10) mg/kg SRF617 or 30 mg/kg (*n* = 11) huIgG4 isotype control Ab. Tumors were measured in two dimensions using a caliper twice per week, and tumor volumes were calculated as volume = (length × width × width)/2.

#### Pharmacokinetics/pharmacodynamics

Thirty-two 5- to 7-wk-old female SCID mice were injected with MOLP-8 cells and divided into seven unblinded groups (six groups with and one control group without treatment, *n* = 3/group) 14 d after tumor inoculation. Mice were treated i.p. with a single dose of 10 mg/kg SRF617, euthanized, and tumor and blood samples were collected at 0, 2, 4, 24, 72, 168, and 336 h. Serum prepared from blood samples was tested for the presence of SRF617 by huIgG4 ELISA (Invitrogen). Tumor samples were split into two. One half was embedded in optimal cutting temperature (OCT) compound and flash frozen, and cryosections were prepared for a CD39 in situ ATPase assay. The other half was dissociated and analyzed by flow cytometry for the presence of SRF617 on MOLP-8 cells using PE-conjugated anti-huIgG4 Ab (clone HP6023; SouthernBiotech). A portion of dissociated cells was spiked with 30 mg/ml SRF617 just prior to staining. The percentage target occupancy on CD39 was determined by comparing the spiked samples, representing 100% occupancy, to the test samples.

#### In situ ATPase assay

In situ ATPase activity on OCT-embedded tissues was performed using a previously published protocol (15). Briefly, OCT-embedded, sectioned tissues were incubated in a medium containing ATP (as substrate) in the presence of other ATPase inhibitors and Pb(NO_3_)_2_ at room temperature for 30 min. Precipitated lead sulfite was imaged for ATPase activity.

### H520 xenograft adenosine measurement

Thirty-five Ncr nu/nu mice were injected s.c. with H520 cells and divided into four unblinded groups when tumors reached 100 mm^3^. Groups in individual cages were treated in the same order i.p. with 15 or 30 mg/kg SRF617 or isotype control (*n* = 10/group) twice per week. Naive mice (*n* = 5) were included to determine baseline levels of adenosine. On day 10, blood was collected, and plasma was isolated and analyzed for adenosine by liquid chromatography tandem mass spectrometry.

### CD39 expression on immune cell subsets after SRF617 treatment

hCD39 KI mice were purchased from Genoway. These mice were developed from a C57BL/6 background by knocking in the mouse CD39 locus with a chimeric CD39 with human extracellular and murine transmembrane and intracellular domains. Female BALB/c hCD39 KI mice were administered 29 mg/kg SRF617 (*n* = 3) or sterile Dulbecco’s PBS (*n* = 3) i.v., and, after 48 h, blood and spleen were collected. Spleen was mechanically dissociated by pushing through a 40-μm nylon mesh filter (Fisher Scientific), and ammonia-chloride-potassium RBC lysis (Thermo Fisher) was performed on resulting splenocyte suspension and isolated blood. Splenocytes and PBMCs were stained with TruStain FcX (BioLegend) for 10 min at 4°C. Samples were stained with viability stain, FITC-conjugated anti-mouse CD19 (clone 65D; BioLegend), Brilliant Violet 421–conjugated anti-mouse CD4 (clone RM4-5; BioLegend), PE-conjugated anti-mouse CD8a (clone 53-6.7; BioLegend), Brilliant Violet 711–conjugated anti-mouse CD11b (clone M1/70; BioLegend), and allophycocyanin-conjugated anti-human CD39 (clone A1; BioLegend) for 30 min at 4°C and analyzed by flow cytometry.

### IHC staining for CD39 and huIgG4 in knock-in mice

Spleens from 12 female BALB/c hCD39 KI mice were isolated 48 h after the mice received a single unblinded i.v. dose of PBS (*n* = 3) or SRF617 (10, 20, or 29 mg/kg [*n* = 3/group]), then formalin fixed and paraffin embedded (FFPE). Dewaxing, Ag retrieval, and staining steps were performed on a Leica Bond Rx instrument. For CD39 staining, FFPE slides were exposed to ER2 solution for 20 min at 100°C for Ag retrieval followed by a 1-h incubation with 63 ng/ml CD39 Ab (clone EPR2046, ab223843; Abcam). For huIgG4 staining, the same Ag retrieval protocol was used, followed by a 1-h incubation with 150 ng/ml anti-huIgG4 Ab (Ab109493; Abcam). Tissues were dehydrated, mounted, and scanned with an Aperio Systems digital scanner. CD39 IHC was quantitated on digitally scanned slides using the Area Quantification module in HALO version 3 (Indica Labs). The result of CD39 IHC quantitation is expressed as the CD39 IHC H-index, which is equal to the percentage of tissue positive for CD39 multiplied by the average OD of tissue positive for CD39 (16).

### CD45 flow cytometric analysis for macrophages

SCID mice were injected s.c. with MOLP-8 cells. When tumors reached 100 mm^3^, mice (*n* = 11/group) were treated i.p. with 20 mg/kg SRF617 or isotype control Ab twice per week for 2 wk. Tumors were collected on day 14 and either formalin fixed (*n* = 3 per group), lysed for cytokine analysis (*n* = 3 per group), or dissociated to single-cell suspensions (*n* = 5 per group). Dissociated tumors were stained with BV421-muCD45, BV711muCD11b, and AF488-muF4/80 for 30 min and analyzed by flow cytometry. The percentage of CD45^+^ cells that was CD11b^+^, F4/80^+^ was gated as macrophages.

### IHC for Ki67, cleaved caspase-3 (CC3), and F4/80 in MOLP-8 xenograft tumors

Unstained FFPE slides were dewaxed and pretreated with Bond Epitope Retrieval 2 solution (Leica Biosystems) for 20 min on a Leica Bond RX autostainer (Leica Biosystems) following the manufacturer’s recommended protocol. DAKO protein block (Agilent Technologies) was applied to slides and incubated for 15 min. Rabbit anti-human Ki67 primary mAb (Abcam) or rabbit anti-CC3 primary mAb (Cell Signaling Technology) was diluted at 0.4 µg/ml concentration (for Ki-67) or 1:400 (for CC3) in Bond Ab diluent (Leica Biosystems), and 150 µl diluted Ab solution was dispensed on each slide and incubated for 60 min at room temperature. Signal was detected by using a Bond Refine Detection kit (Leica Biosystems). Briefly, slides were incubated with anti-rabbit-HRP polymer for 8 min, with diaminobenzidine for 10 min, and with diaminobenzidine enhancer for 10 min. Slides were counterstained with hematoxylin for 20 min, followed by bluing reagent incubation for 3 min. Slides were manually dehydrated, coverslipped with SignalStain Mounting Medium (Cell Signaling Technology), and digitally scanned at 20× using an Aperio Versa 200 scanner (Leica Biosystems). Quantitative image analysis was performed using the Multiplex IHC algorithm version 3.2.3 (for Ki-67) and Area Quantification algorithm version 2.4.2 (for CC3) in HALO version 3.5 (Indica Labs). The Tissue Classifier algorithm was used to exclude areas of necrosis. The proliferation index indicates the percentage of Ki-67-positive cells. The CCR3 H-index indicates the percentage of tissue that is positive for CC3 multiplied by the average OD of the tissue that is positive for CC3.

F4/80 staining on fixed and sectioned MOLP-8 xenograft tumors was performed with rat anti-mouse F4/80 (Bio-Rad Laboratories). Slides were scanned on an Aperio AT2 whole-slide scanner. Image analysis on digital slide images with Visiopharm software was used to determine F4/80 expression as a percentage positive value within the tumor area region of interest.

### Murine tumor cytokine analysis

For the cytokine analysis, a portion of each tumor was lysed and homogenized with radioimmunoprecipitation assay lysis and extraction buffer (Thermo Fisher) containing Halt protease inhibitor mixture (Thermo Fisher) in a Fast Prep tissue homogenizer (MPBio). After lysis and homogenization, protein concentration was determined by using a bicinchoninic acid assay kit (Thermo Fisher). Each lysate (1 μg/well) was analyzed for murine MCP-1, MIP-1α, and MIP-1β by MSD multiplex assay.

### KPC orthotopic model SRF617 staining and CD8^+^ tumor-infiltrating lymphocyte analysis

KPC 1245 pancreatic tumor cells (KPC cells) were a kind gift from Prof. David A. Tuveson (Cold Spring Harbor Laboratory). KPC cells were stably transfected by lentiviral vectors containing pCDH-EF1-cOVAv2-T2A-copGFP (donated by Prof. Jonathan Weissman, Stanford University) and CSCW-Gluc-CFP (donated by Dr. Bakhos A. Tannous, Massachusetts General Hospital) to create a tumor cell line expressing OVA and secreting *Gaussia* luciferase (KPC OVA Gluc). Tumor cells were grown in DMEM supplemented with 10% FBS and 1% penicillin/streptomycin. Cells were maintained at 37°C in an incubator with a controlled humidified atmosphere composed of 95% air and 5% CO_2_.

For orthotopic tumor injections, KPC cells were resuspended in PBS and Corning Matrigel Matrix High Concentration at a ratio of 1:1. A total of 50,000 cells in 10 μl were injected into the pancreas of twenty 8- to 10-wk-old female hCD39 KI mice. Mice received four unblinded doses of SRF617 (30 mg/kg, *n* = 10) or huIgG4 isotype control Ab (*n* = 10) by i.p. injections every 3 d starting on day 4. Tumor growth was monitored by measuring the concentration of secreted Gluc in the blood (17). Pancreatic tumors were harvested 15 d after orthotopic tumor implantation and cut in half. One half was processed for FACS analysis, and the other was fixed in 10% formaldehyde for IHC staining for huIgG4.

Tumors for flow analysis were cut with scissors and subsequently digested using collagenase type IV and DNase I. Cell suspensions were filtered using a 70-µm cell strainer and resuspended in PBS, 2% FBS, 5 mM EDTA (FACS buffer). Following Fc block (anti-CD16/32 mAb for 15 min at 4°C), Abs against cell surface Ags were added (CD45 and CD8) (30 min at 4°C) in Brilliant Stain Buffer (BD Biosciences), followed by viability staining in PBS. Fluorescence minus 1 controls were always included as negative controls. Flow cytometry was performed on an LSRFortessa X-20 device (BD Biosciences), and FlowJo software was used for analysis.

## Results

### SRF617 binds to human CD39 and inhibits ATPase activity

**FIGURE 1.**
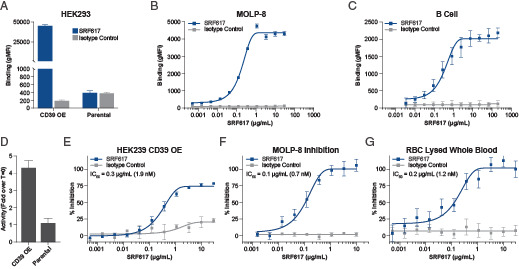
SRF617 binds to and inhibits cellular CD39. The figure legend refers to all the depicted graphs. (**A**) HEK293 OE and parental HEK293 cells were cultured with SRF617 or huIgG4 isotype control antibody (both 10 μg/ml) for 1 h, followed by incubation with PE-conjugated anti-huIgG4 secondary antibody. Cells were analyzed for SRF617 binding by flow cytometry. (**B** and **C**) MOLP-8 or isolated primary human B cells were incubated with SRF617 or huIgG4 isotype control antibody (dose range shown) for 1 h followed by staining with PE-conjugated anti-huIgG4 secondary antibody. Binding of SRF617 was assessed by flow cytometry. (**D**) HEK293 OE or parental control cells were incubated with 25 µM ATP and incubated for 1 h. ATP hydrolysis was measured using the malachite green assay. Relative activity is shown as fold over T = 0 condition. (**E**–**G**) HEK293 OE cells, MOLP-8 cells, or RBC-lysed whole blood were pretreated with SRF617 for 1 h and then 25 µM ATP. ATP hydrolysis was measured using the malachite green assay, and percentage inhibition was determined. In each graph, error bars represent a range of duplicates, and the experiment is representative of similar results repeated at least three times. gMFI, geometric mean fluorescence intensity.

SRF617 was discovered using a yeast Ab display selection campaign. After multiple rounds of selection using biotinylated recombinant human CD39 on yeast cells expressing a diverse library of human Abs, SRF617 was selected from a panel of Abs with high-affinity binding to human CD39. Analysis using BLI for SRF617 demonstrated high-affinity binding to recombinant human and cynomolgus monkey CD39, but not murine CD39 (Table I). To assess binding of SRF617 to cellular CD39, flow cytometric analysis was performed on cells expressing high levels of surface CD39 incubated with various concentrations of SRF617. SRF617 binds specifically to HEK293 cells engineered to overexpress full-length human CD39 (HEK293 OE) as compared with lack of binding to the parental HEK293 cells and huIgG4 isotype control Ab (Fig. 1A). SRF617 binds in a specific dose-dependent manner to primary human B cells and the human multiple myeloma cell line MOLP-8 (Fig. 1B, 1C).

**Table I.** Summary of SRF617 binding to recombinant human, cynomolgus monkey, and murine CD39

Recombinant Analyte	Ligand	*K*_D_ (M)	*k*_d_ (1/M·s)	*k*_a_ (1/s)	Full χ^2^	Full *R*^2^
Human CD39	SRF617	1.11E-09	5.19E-04	4.67E+05	0.5093	0.9878
Cynomolgus monkey CD39	SRF617	4.06E-10	3.05E-04	7.51E+05	0.3215	0.9953
Murine CD39	SRF617	Not bound	NA	NA	NA	NA

*R*^2^ values >0.95 and χ^2^ values <3.0 demonstrate good model fit.

Note: Murine CD39 binding assay was qualified using a rat anti-murine CD39 Ab (Duha59) as positive control.

Ab-mediated inhibition of ATPase activity was assessed using a malachite green assay to detect phosphate released from ATP hydrolysis. HEK293 OE cells have increased enzymatic activity compared with their parental counterparts (Fig. 1D). SRF617 inhibited the enzymatic ATPase activity of these cells in a concentration-dependent manner (IC_50_ of 0.3 μg/ml [1.9 nM]), whereas an isotype control Ab did not (Fig. 1E). Similar anti-ATPase activity for SRF617 was observed on MOLP-8 tumor cells (IC_50_ of 0.1 μg/ml [0.7 nM]) and in PBMCs from whole blood (IC_50_ of 0.2 μg/ml [1.2 nM]) (Fig. 1F, 1G). In additional experiments, CD39 inhibition by SRF617 was shown to be noncompetitive. Equivalent inhibition was observed with a range of ATP present (Supplemental Fig. 1).

### SRF617 enhances CD4^+^ T-cell proliferation in the presence of exogenous ATP

**FIGURE 2.**
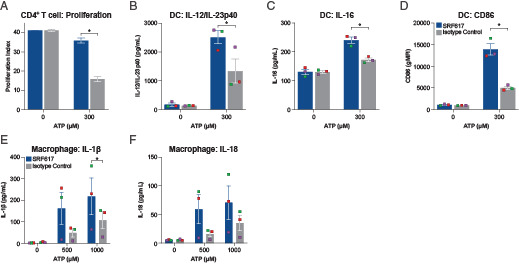
SRF617 rescues CD4^+^ T-cell proliferation and enhances both DC maturation and inflammasome activation of macrophages in the presence of ATP. The color code in the figure legend refers to all the depicted bar graphs. (**A**) Pooled CD4^+^ T cells from three donors were prestained with CellTrace Violet and incubated with SRF617 or isotype control antibody, 250 μM ATP, and Dynabeads (T-cell activator) for 3 d. Proliferation was analyzed by flow cytometry. The proliferation index, a measure of the extent of proliferation of the T cells, was calculated using the CellTrace Violet flow data and graphed as shown. Error bars represent the SD of three replicate results run on the same plate with a donor pool. Experiment is representative of results repeated at least three times. (**B** and **C**) Monocyte-derived DCs from one donor were treated for 1 h with 10 μg/ml SRF617 or isotype control antibody with or without 300 μM ATP for 48 h. Supernatants were collected and analyzed for IL-12/IL-23p40 or IL-16 by MSD. Error bars represent SD of three replicates run on the same plate. (**D**) Monocyte-derived DCs from three donors were treated for 1 h with 10 μg/ml SRF617 or isotype control antibody with or without 300 μM ATP for 24 h. Cells were collected, stained with PE-CD86, and analyzed by flow cytometry. (**E** and **F**) Monocyte-derived macrophages from three different donors were pretreated with 10 µg/ml SRF617 or isotype control antibody for 1 h and subsequently treated with 10 ng/ml LPS for 3 h. Afterward, cells were treated for 2 h with 0, 500, or 1000 µM ATP, and supernatant was collected and analyzed for IL-1β (E) and IL-18 (F) by MSD. For (B–F), data are from three separate donors, and each donor is represented by a different color dot. Error bars represent the SD of the three separate donor results. *p < 0.05 determined by two-tailed paired *t* test. Results are representative, and experiments were repeated at least three times. gMFI, geometric mean fluorescence intensity.

Expression of both CD39 and CD73 on CD4^+^ T cells isolated from PBMCs has been described previously (18, 19). Exogenous ATP, when not degraded, increases proliferation of activated CD4^+^ T cells, but cellular CD39 hydrolyzes exogenous ATP to AMP. AMP is further reduced to adenosine, leading to potent suppression of the proliferation response (20). Therefore, inhibition of CD39 after exogenous addition of ATP should lead to maintenance of ATP levels and reduce the production of adenosine, leading to a stimulatory effect and a loss of suppression on CD4^+^ T cells. To examine the potential of SRF617 to inhibit CD39 and enhance T-cell proliferation in the presence of ATP, a flow cytometry assay of T-cell proliferation was used. Briefly, isolated CD4^+^ T cells were stimulated with anti-CD3/CD28 beads in the presence or absence of ATP and SRF617. Results from three individual donors showed that SRF617 enhanced the proliferation index of CD4^+^ T cells in the presence of ATP by roughly 2-fold (Fig. 2A) compared with an isotype control Ab, demonstrating that SRF617-driven CD39 inhibition prevented the expected decrease in T-cell proliferation caused by ATP reduction to adenosine in vitro.

### SRF617 enhances dendritic cell maturation in the presence of ATP

Markers of DC maturation, such as CD86 expression, have been shown to increase with exogenous ATP stimulation (21). DCs are known to express high levels of CD39 that can rapidly convert ATP to AMP, reducing these ATP-induced effects (21–23). To assess the functional outcome of SRF617-induced CD39 inhibition on DCs in vitro, three markers of DC maturation (CD86 expression, IL-12/IL-23p40 secretion, and IL-16 secretion) were examined on monocyte-derived DCs isolated from buffy coats of individual human donors. SRF617 raised the levels of CD86 on the surface of DCs and induced higher levels of IL-16 and IL-12/IL-23p40 secretion in the presence of ATP, indicating that SRF617 enhanced DC maturation and activity in vitro (Fig. 2B–2D).

### SRF617 shows evidence of enhanced inflammasome activation of macrophages in vitro

Extracellular ATP, in combination with LPS, is known to induce inflammatory responses in macrophages through activation of P2RX7 and subsequent induction of NLRP3 inflammasomes (24). Previous studies have also shown that inhibition of CD39 on macrophages can enhance inflammasome response by elevating or maintaining high extracellular ATP levels, which leads to release of IL-1β and IL-18 (25). To demonstrate the ability of SRF617 to increase inflammasome activity, levels of IL-1β and IL-18 were measured in supernatants from macrophages exposed to LPS and ATP (Fig. 2E, 2F).

### SRF617 reduces CD39 enzymatic activity in vivo and has antitumor effects in MOLP-8 and H520 xenograft tumor models

**FIGURE 3.**
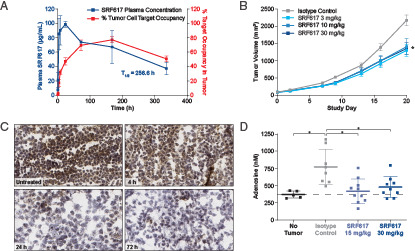
SRF617 shows single-agent activity in two xenograft models, inhibits ATPase activity in the TME, and reduces elevated adenosine levels in tumor-bearing mice. (**A**) SCID mice were injected s.c. with MOLP-8 tumor cells. On day 14, mice with tumors were treated once i.p. with 10 mg/kg SRF617 (*n* = 3/time point). At the indicated time points, plasma SRF617 levels were determined by ELISA (blue line). Tumors were dissociated into single-cell suspensions, split, and dosed with 1 mg/ml SRF617 and PE-conjugated anti-huIgG4 or directly stained with PE-conjugated anti-huIgG4. The percentage tumor occupancy was calculated using flow cytometry. The percentage tumor occupancy (mean ± SEM) from cells treated with PE-conjugated anti-huIgG4 at the indicated time points is shown (red). (**B**) SCID mice were injected s.c. with MOLP-8 cells. When tumors reached ∼100 mm^3^, mice were treated i.p. with 3, 10, or 30 mg/kg SRF617 or isotype control antibody (*n* = 10 or 11/group) twice per week for 3 wk. Data are shown as mean ± SEM. *p < 0.0001 determined by two-tailed unpaired *t* test. (**C**) SCID mice were injected s.c. with MOLP-8 cells. On day 14, mice (*n* = 3/group) were treated once i.p. with 10 mg/kg SRF617 or left untreated. After 4, 24, and 72 h, tumors were collected and embedded in OCT compound, and a CD39 in situ ATPase assay was performed on cryostat tissue sections. Representative sample images from different treatment groups are shown (original magnification ×20). Brown stain represents ATPase activity. (**D**) Ncr nu/nu mice were injected s.c. with H520 cells. When tumors reached 100 mm^3^, mice (*n* = 10/group) were treated i.p. with 15 or 30 mg/kg SRF617 or isotype control twice per week. Naive mice (*n* = 5) were included to determine the baseline level of adenosine. On day 10, blood was collected, and plasma was isolated and analyzed for adenosine by liquid chromatography-tandem mass spectrometry. Data are shown as mean plasma adenosine ± SEM. *p < 0.05 determined by two-tailed unpaired *t* test. Pharmacokinetic properties, target engagement, and in situ enzymatic inhibition experiments are representative of results repeated at least two times.

To examine the antitumor activity of SRF617 via inhibition of CD39 enzymatic activity in the TME, a MOLP-8 xenograft s.c. tumor model was used. The MOLP-8 model was chosen due to high levels of human CD39 expression on MOLP-8 cells. Flow cytometric analysis to examine SRF617 bound to CD39 on isolated, dissociated tumor cells demonstrated increased target occupancy concomitant to Ab serum levels correlating well with the half-life of SRF617 of 256.6 h (Fig. 3A). SRF617 significantly reduced MOLP-8 tumor growth compared with an isotype control Ab (Fig. 3B). In addition, the use of an in situ enzymatic activity assay in tumor sections from mice treated with SRF617 demonstrated strong inhibition of ATPase activity in the TME within 24 h (Fig. 3C).

**FIGURE 4.**
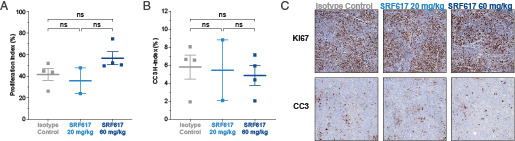
SRF617 does not modulate proliferation or apoptosis in MOLP-8 xenograft tumors. SCID mice were injected s.c. with MOLP-8 cells. When tumors reached 100 mm^3^, mice (*n* = 2–4/group) were treated i.p. with 20 mg/kg SRF617, 60 mg/kg SRF617, or isotype control antibody twice per week for 2 wk. Tumors were collected on day 14 and formalin fixed. Fixed samples were stained for (**A**) Ki-67 or (**B**) CC3. The proliferation index (%) was calculated from Ki-67–stained images, and the CC3 H-index was quantitated. Data are shown as mean ± SEM. (**C**) Representative images from the treatment groups are shown (original magnification ×10).

The ability of SFR617 to lower levels of circulating adenosine in tumor-bearing mice was assessed using the H520 xenograft model, in which high levels of CD39 are expressed on H520 cells. Significantly higher levels of circulating adenosine were detected in tumor-bearing versus naive mice. SRF617 significantly decreased plasma adenosine levels in tumor-bearing mice compared with isotype control Ab–treated animals (Fig. 3D). Quantitation of cellular proliferation and apoptosis in the MOLP-8 tumors was examined by quantitation of IHC staining for the markers Ki-67 and CC3, respectively. No significant differences in proliferation or apoptosis were observed in xenograft tumor sections from tumors treated with two different dose levels (Fig. 4). In addition, in vitro experiments to examine the potential for direct effect of SRF617 on MOLP-8 cell proliferation were performed. Treatment of MOLP-8 cells with SRF617 in vitro did not modulate their proliferation (Supplemental Fig. 2).

### SRF617 induces macrophage infiltration in the MOLP-8 xenograft model

**FIGURE 5.**
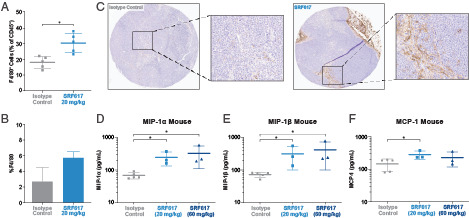
SRF617 increases macrophage infiltration in MOLP-8 xenograft tumors. SCID mice were injected s.c. with MOLP-8 cells. When tumors reached 100 mm^3^, mice (*n* = 10 or 11/group) were treated i.p. with 20 mg/kg SRF617, 60 mg/kg SRF617, or isotype control antibody twice per week for 2 wk. Tumors were collected on day 14 and either formalin fixed (*n* = 2–4) or dissociated to single-cell suspensions (*n* = 5). (**A**) Dissociated tumors from the isotype control or 20 mg/kg SF617 groups were stained with BV421-muCD45, BV711-muCD11b, and AF488-muF4/80 for 30 min and analyzed by flow cytometry. The percentage of CD45^+^ cells that was CD11b^+^, F4/80^+^ was gated as macrophages. (**B**) Fixed samples were stained for muF4/80, and a percentage positive pixel (mean ± SEM) algorithm was used for quantitation. Data shown are mean ± SEM. *p < 0.05 determined by two-tailed unpaired *t* test. (**C**) muF4/80 stained Sample images from the different treatment groups are depicted (original magnifications ×2 and ×10). (**D**–**F**) Tumor lysates collected on day 14 (*n* = 3–5) were prepared and analyzed for murine cytokine production using MSD immunoassays for MIP-1α, MIP-1β, and MCP-1. Data shown are the mean ± SD cytokine levels from five isotype control and three SRF617-treated tumor lysates. Individual mouse samples are represented by dots, squares, and triangles. *p < 0.05 determined by two-tailed paired *t* test.

To better understand SRF617-driven tumor growth inhibition, we characterized immune cell infiltration in the TME. MOLP-8 xenograft tumors were examined by IHC and flow cytometry for the presence of CD45^+^ cells and F4/80 macrophages following treatment. Flow cytometric analysis of dissociated tumors from SRF617-treated animals revealed significantly higher levels of CD45^+^ immune cell infiltration compared with isotype control Ab–treated tumors (Fig. 5A). IHC imaging revealed increased staining of F4/80 macrophages in the tumors of SRF617-treated mice (Fig. 5B, 5C). To elucidate the mechanism of macrophage recruitment, tumor lysates were prepared and analyzed for murine cytokine levels by MSD. Murine macrophage-attracting cytokines MIP-1α, MIP-1β, and MCP-1 were all significantly elevated in the SRF617-treated tumors compared with isotype controls (Fig. 5D–5F).

### SRF617 reduces the expression of CD39 on immune cell subsets

**FIGURE 6.**
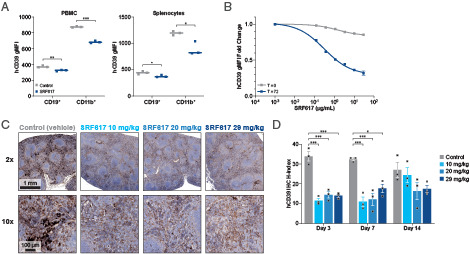
SRF617 treatment reduces CD39 expression on immune cells. (**A**) hCD39 KI mice were dosed once i.v. with 29 mg/kg SRF617. After 48 h, PBMCs from whole blood and splenocytes were isolated, stained, and analyzed by flow cytometry to measure CD39 expression (geometric mean fluorescence intensity [gMFI]) on CD19^+^ and CD11b^+^ cells. (**B**) Cryopreserved PBMCs isolated from human whole blood were treated with SRF617 (concentrations shown) for 72 h or immediately prior to analysis (T = 0). Cells were stained and analyzed by flow cytometry to measure CD39 expression (gMFI) on CD19^+^ cells depicted as fold change from untreated cells. (**C** and **D**) hCD39 KI mice were dosed once i.v. with 10, 20, or 29 mg/kg SRF617 and spleens were harvested on days 3, 7, and 14 and processed for hCD39 IHC stain (*n* = 3). (C) Representative images from day 3 spleens at low (2×) and high (10×) magnification. (D) hCD39 IHC was quantified on digitally scanned slides using the area quantification module of the HALO imaging analysis suite. H index = [percentage of tissue positive for hCD39] × [average optical density of tissue positive for hCD39]. Data are presented as mean ± SEM. Dots indicate individual mice. *p < 0.05, ***p < 0.001 as determined by two-tailed unpaired *t* test. The human B cell result is representative of two separate experiments in different donors. In vivo experiments in hCD39 KI mice exploring SRF617 modulation of CD39 expression are representative of two separate experiments.

To further characterize the mechanistic effects of SRF617 in syngeneic mouse tumor models, hCD39 KI mice were used. After a single dose of SRF617, flow cytometry revealed significant reduction of CD39 expression on circulating CD19^+^ and CD11b^+^ cells and the same cells from isolated spleens (Fig. 6A). Dose-dependent reduction of CD39 expression was similarly observed on CD19^+^ B cells from human PBMCs after treatment with SRF617 (Fig. 6B). The T = 0 cross-blocking control curve demonstrates that the flow Abs for all flow studies that were used do not significantly interfere with each other’s binding. No change in viability of B cells was observed. To further investigate the reduction of CD39 expression, spleens from hCD39 KI mice treated with a single dose of SRF617 were harvested 3, 7, and 14 d after administration and analyzed by IHC for CD39 expression. Significant decreases in CD39 expression were observed for all doses tested on days 3 and 7. At 14 d, CD39 expression had recovered, especially at the lower 10 mg/kg dose (Fig. 6C, 6D).

### SRF617 penetrates the TME and increases CD8^+^ T-cell infiltration in orthotopic KPC tumors

**FIGURE 7.**
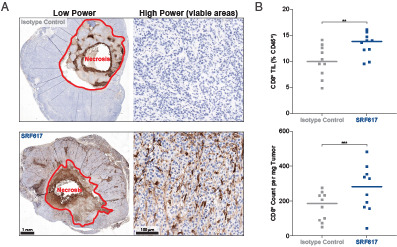
SRF617 penetrates the TME and increases CD8^+^ T-cell infiltration in orthotopic KPC tumors. hCD39 KI mice implanted orthotopically with KPC tumors received four doses of 30 mg/kg SRF617 or huIgG4 isotype control antibody by i.p. injections every 3 d starting on day 4. Pancreatic tumors were harvested 15 d after orthotopic tumor implantation and cut in half. One half was processed for FACS analysis, and the other was fixed in 10% formaldehyde for IHC staining. (**A**) IHC staining for huIgG4 was performed on FFPE orthotopic KPC tumors from mice treated with 30 mg/kg isotype control antibody (top panels) or SRF617 (bottom panels). Scale bars indicate 1 mm (low-power panels [original magnification ×0.6], left) and 100 µm (high-power panels [original magnification ×10], right). Staining is representative of three tumor samples from both isotype control– and SRF617-treated tumors. (**B**) Dissociated tumors were stained and analyzed by FACS to measure infiltration of CD8^+^ T cells. Data are shown as both the percentage of total CD45^+^ cells and CD8^+^ T cell count per milligram of tumor. **p < 0.01, ***p < 0.001 as determined by two-tailed unpaired *t* test. Each dot in the graphs represents data from separate mouse tumor samples.

To observe the ability of SRF617 to achieve exposure in an orthotopic tumor setting, hCD39 KI mice were implanted orthotopically in the pancreas with KPC tumor cells and treated with multiple doses of SRF617 or isotype control Ab. The resulting tumors were examined 15 d after implantation by IHC to observe SRF617 staining (huIgG4) and analyzed by flow cytometry to measure CD8^+^ T-cell infiltration. The orthotopic tumors showed strong SRF617 staining in the viable tumor regions, as evidenced by IHC signal for huIgG4, whereas nonspecific isotype control Ab staining was observed only in the necrotic core (Fig. 7A). Flow cytometric analysis of dissociated tumor revealed that SRF617 induced a significantly higher level of CD8^+^ T-cell tumor infiltration than isotype control Ab when measured as CD8^+^ T cells as a percentage of total immune infiltrate or absolute count per milligram of tumor (Fig. 7B).

## Discussion

Targeting the adenosine pathway in combination with other therapies is emerging as an attractive approach to treating cancer. Investigational drugs in development include inhibitors of major components in the pathway (i.e., the ectoenzymes CD39 and CD73 and adenosine receptors A2a/A2b). Multiple clinical studies with CD73 inhibitors and adenosine receptor antagonists have shown positive clinical outcomes as monotherapy and in combination with chemotherapy and/or immune checkpoint inhibitors (7–12). In a randomized phase 2 study in non–small cell lung cancer, the anti-CD73 Ab oleclumab in combination with anti-PD-L1 agent durvalumab provided a significant improvement in progression-free survival over durvalumab alone (hazard ratio, 0.44) (26). These results demonstrate great potential for targeting adenosine production in the TME to relieve immune suppression. Although targeting CD73 or adenosine receptors can attenuate immune suppression in the TME, targeting of CD39 provides an additional immune-stimulatory effect by stabilizing ATP levels in tumors. This study describes the mechanism of action and antitumor potential of SRF617. SRF617 inhibits CD39 ATPase activity in the TME, which concomitantly stabilizes ATP to increase inflammatory response and lowers adenosine to remove immune suppression.

High levels of ATP (up to millimolar amounts) can be produced in the TME due to cellular stress, cytotoxicity, and cell damage (2, 27). Extracellular ATP, a potent “danger signal” acting mostly through the P2RX7 purinergic receptor on immune cells, stimulates immune responses on multiple cell types, primarily those of myeloid lineage (28–31). P2RX7 receptors have a relatively low affinity for ATP, so the inflammation response occurs only if high levels of ATP are maintained. CD39 is present at high levels in many types of tumors and can rapidly convert ATP to AMP, contributing to immune evasion by lowering ATP levels. We demonstrate that SRF617 can potentiate ATP effects in vitro in two ways: by stimulating maturation and cytokine secretion of DCs and by enhancing the release of IL-1β and IL-18 from macrophages. ATP-activated DCs have been shown to activate cytotoxic T cells more potently (31). Release of the cytokines IL-1β and IL-18 after exposure to extracellular danger signals initiates a powerful inflammatory response that leads to recruitment of immune cells, pyroptosis, and potentiation of CD8^+^ T cell effector function (28). In tumors, ATP signaling through P2RX7 and NALP3 brought about by inhibiting CD39 has been shown to stimulate these responses in the TME and is intrinsic to the antitumor activity by blocking CD39 enzymatic activity (32). In the MOLP-8 xenograft model, we show a large increase in tumor macrophage infiltration upon treatment with SRF617 associated with SRF617 binding to CD39 on MOLP-8 cells and inhibition of CD39 ATPase activity. ATP is a potent chemoattractant for macrophages, making it likely that these cells are responding to an increased steady-state level of ATP (29). In addition, in tumors treated with SRF617, we observed an increase of murine cytokines that attract macrophages (MCP-1, MIP-1α, and MIP-1β). Given that the cytokines detected are murine proteins, they are likely originating from the stromal compartment within the TME and are indicative of an enhanced state of inflammation. ATP can also modulate the function of other immune cells, including CD4^+^ and CD8^+^ T cells, by boosting proliferation and inducing chemotaxis (2, 27, 28, 31, 33). Finally, ATP has been shown to enhance metabolism in memory CD8^+^ T cells (33). We demonstrate that SRF617 enhances CD4^+^ T-cell proliferation in the presence of ATP and induces higher numbers of CD8^+^ T cells in the TME of an orthotopic pancreatic tumor in vivo. The increase in CD8^+^ T cells in the orthotopic tumor model could be attributed to either elevated ATP levels causing increased chemotaxis or a decrease in adenosine causing less immune suppression in the TME or both. At a minimum, it suggests that inhibition of CD39 in the TME is leading to a higher state of inflammation. Further studies into the exact mechanism for increased CD8^+^ T cell infiltration will be required to fully understand this phenomenon.

On the other end of the pathway, adenosine is a potent immune suppressor with wide-reaching effects on multiple immune cell types, including T, NK, and myeloid cells (20). Reduction of CD4^+^ T-cell proliferation by ATP is driven mostly by the rapid production of adenosine and subsequent signaling via the inhibitory A2aR. T-cell proliferation can also be suppressed by AMP or adenosine and can be reversed by CD73 enzymatic inhibition or adenosine receptor antagonism (20). We show that SRF617 can reduce the production of adenosine from ATP and rescue proliferation of CD4^+^ T cells in the presence of ATP. Furthermore, we demonstrate that SRF617 can lower systemic adenosine levels in the H520 tumor model, where adenosine levels are elevated by the presence of tumor.

We show that SRF617 is a potent inhibitor of the ATPase activity of CD39 and observe that SRF617 can lower expression of CD39 on immune cells, further lessening its effectiveness as an ATP degrader. Loss of cell surface CD39 was observed in circulating immune cells from SRF617-treated hCD39 KI mice and on ex vivo cultured primary human CD19^+^ B cells that cannot be attributed to detection Ab cross-blocking or cellular toxicity. It is also unlikely that SRF617 could cause significant Ab-mediated cell death because it is an Fc silent IgG4 Ab. Loss of CD39 expression on B cells and other CD39-expressing cells is a phenomenon also observed in an ongoing SRF617 phase 1 clinical study (NCT04336098). In humans treated with SRF617, a dose-dependent reduction of CD39 was observed on circulating B cells correlating with CD39 target occupancy. In addition, a reduction in CD39 expression was observed in matched tumor biopsies from before and after SRF617 treatment (34).

In murine tumor models (s.c. and orthotopic), SRF617 penetrates the TME and shows high levels of exposure that correlate with serum levels. We observed inhibition of the in situ enzymatic activity of CD39 correlating with maximal SRF617 occupancy in MOLP-8 tumors. In an orthotopic KPC tumor model study conducted in hCD39 KI mice, we observed stromal IHC staining for huIgG4 in the viable compartment of the TME of SRF617-treated tumors and no staining in the TME of isotype control–treated tumors. The presence of huIgG4 in these tumors can be attributed only to the presence of SRF617. Although staining is observed in the necrotic compartments of both treatment groups, this is likely due to nonspecific staining that is common in necrotic tissue. Taken together, these results provide strong evidence for SRF617 tumor exposure, enzymatic inhibition, and increased tumor immune cell infiltration in the TME.

The properties of SRF617 make it an excellent drug development candidate targeting the ectoenzyme CD39. Furthermore, by targeting the first enzyme in the ATP-degrading adenosine pathway, and thereby both maintaining high levels of ATP and reducing levels of adenosine in the TME, SRF617 has great potential for cancer treatment, especially in combination with immunogenic agents or other immuno-oncology–based treatments.

## Supplementary Material

Supplemental Figures 1 (PDF)
