# Supplementary material for: SRF617 Is a Potent Inhibitor of CD39 with Immunomodulatory and Antitumor Properties
Source: Immunohorizons. 2023 May 23;7(5):366–79. doi: 10.4049/immunohorizons.2200089 (PMC10579980; doi:10.4049/immunohorizons.2200089)
Supplement: Supplemental Figures 1 (PDF) [file IH_2200089_Supplemental_1.pdf]

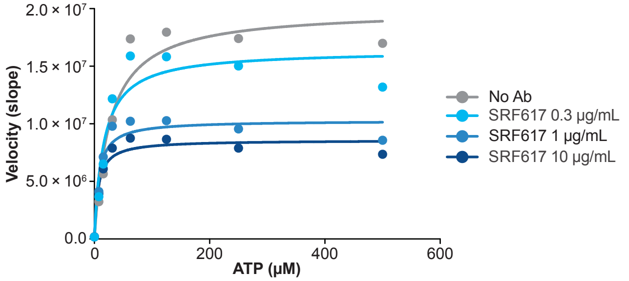

Supplemental Figure S1: SRF617 is a non-competitive inhibitor of CD39
Recombinant human CD39 (4.4 μg/μL) was preincubated ± SRF617 at the concentrations indicated. ATP substrate was added as a titration from 500 – 7.8 μM including a zero ATP well. ATP hydrolysis was measured using an enzyme-coupled reaction with Amplex red as the indicator. Fluorescent readouts (ex/em 540/590) were collected kinetically each minute for 25 minutes, and rate (velocity) of ATP conversion was determined for each condition. Michaelis-Menten kinetics curves (rate vs substrate concentration) were generated as shown. K_m determination was done as close to initial steady-state conditions as possible for the assay. Increasing concentrations of SRF617 lowered the V_{max} the while having little impact on the K_m. The Amplex red assay was performed as described in Helenius M., Jalkanen S., Yegutkin G. 2012. Enzyme-coupled assays for simultaneous detection of nanomolar ATP, ADP, AMP, adenosine, inosine and pyrophosphate concentrations in extracellular fluids. Biochim Biophys Acta. 1823(10):1967-1975.

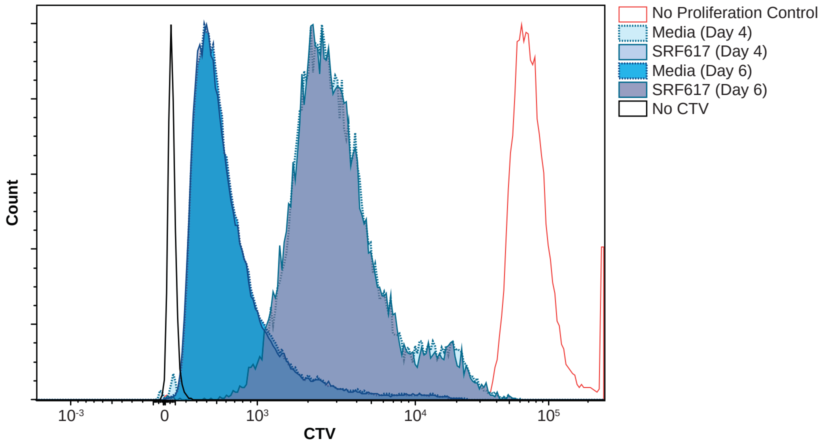

Supplemental Figure S2: SRF617 does not modulate MOLP-8 cell proliferation in vitro
MOLP-8 cells were stained with CellTrace Violet (CTV) according to the manufacturer's instructions and seeded into a 96-well plate at 5 × 10^4 cells/well. Cells were treated with media alone or media containing SRF617 and incubated to allow proliferation for 0, 4, and 6 days. Cells were analyzed by flow cytometry to determine the extent of CTV staining. Day 0 cells and unstained MOLP-8 cells were analyzed as controls. The graphs are histograms depicting the extent of CTV stain in the 6 conditions as noted by the figure legend. Media alone and SRF167-treated peaks are almost completely overlapping at days 4 and 6 demonstrating no difference in cellular proliferation between these conditions.
